# Fast and Fault-Tolerant Passive Hyperbolic Localization Using Sensor Consensus

**DOI:** 10.3390/s24092891

**Published:** 2024-04-30

**Authors:** Gyula Simon, Gergely Zachár

**Affiliations:** 1 Alba Regia Technical Faculty, Óbuda University, 8200 Székesfehérvár, Hungary; 2 Institute for Software Integrated Systems, Vanderbilt University, Nashville, TN 37212, USA; gergely.zachar@vanderbilt.edu

**Keywords:** source localization, emitter localization, TDOA, fault tolerant, consensus function

## Abstract

The accuracy of passive hyperbolic localization applications using Time Difference of Arrival (TDOA) measurements can be severely compromised in non-line-of-sight (NLOS) situations. Consensus functions have been successfully used to provide robust and accurate location estimates in such challenging situations. In this paper, a fast branch-and-bound computational method for finding the global maximum of consensus functions is proposed and the global convergence property of the algorithm is mathematically proven. The performance of the method is illustrated by simulation experiments and real measurements.

## 1. Introduction

Determining the location of an object is a key service in a wide range of applications, including navigation [1,2], robotics [3,4], tracking [5,6], safety and security [7,8], healthcare [9,10], and defense [11,12], just to name a few. Global Navigation Satellite Systems (GNSSs) can be used in various outdoor applications [1], while for indoor and other GNSS-deprived areas, several other technologies have been proposed, based on, e.g., acoustic signals [8,11,12,13], radio signals [5,9], visible light [4,14], or images [15,16].

The localization systems utilize a number of spatially distributed anchors, and most often measure quantities directly related to distances, distance differences, angles, or angle differences between the anchors and the target. The location estimate is then calculated using Time of Arrival (TOA), Time Difference of Arrival (TDOA), angle of arrival (AOA), or angle difference of arrival (ADOA) methods, respectively [17]. 

The TDOA localization, also known as hyperbolic positioning, is a fairly common method in geolocation [1,18,19], sensor networks [8,11,20,21], indoor localization [22,23], or the Internet of Things [24,25]. In the TDOA scenario, differences of distances between the target and the anchors are used, most often calculated from differences of measured times of arrivals of signals (sound or electromagnetic waves) between the anchors and the target, using the propagation speed of the signal. The one-stage or Direct Position Determination (DPD) methods determine the position estimate without explicitly calculating the Time Difference of Arrival (TDOA) values. In contrast, the two-stage methods estimate the TDOA values in stage 1 and then estimate the location from them in stage 2. The location estimation process is also known as position fixing. Although two-stage methods are not optimal, they are asymptotically equivalent to DPD methods and are popular in systems where the transmission of large amounts of raw data between sensors and the central processing unit is prohibitive [26]. This paper focuses on the position fixing of two-stage methods.

The process of position fixing most often involves closed-form solutions [18,27,28,29,30,31,32], iterative techniques [33,34], or various consensus-based methods [35,36,37,38,39,40]. These methods yield satisfactory results when the time difference measurements contain only small errors (i.e., measurement noise). However, in practical scenarios, measurements can contain significant errors (outliers), especially when there is no direct line of sight between the anchors and the target, or when the environment produces signal reflections, or when signal detection, and thus time measurements, is not reliable. If such outlier measurements are present, the position estimates of closed-form and iterative techniques will be biased unless additional measures are taken to remove the outliers prior to the estimation [41,42]. Consensus-based methods can provide accurate estimates even when outlier measurements are present, and were successfully utilized in applications where non-line-of-sight conditions and reflections cause unreliable measurements [38,39]. 

In this paper, a consensus-based method is studied, where the position estimate is determined by identifying the location with the highest value of the consensus function [38]. A fast computation method was recently proposed that was observed to reliably find the global optimum of the consensus function [40]. This paper reviews the consensus function and introduces an updated search method. The contributions of this paper are the following:

- A new, fast evaluation method is introduced.
- The main contribution is the theoretical proof that ensures that the proposed algorithm always finds the global maximum of the consensus function over a finite grid.
- Finally, a comprehensive performance analysis is provided using simulations and real measurements.

The symbols used in this paper are enumerated in the Section: List of Symbols.

## 2. Related Work

In Section 2.1, the TDOA problem is formulated, with special emphasis on potential large measurement errors, e.g., due to non-line of sight (NLOS). The consensus-function-based solution is reviewed in Section 2.2, and various fast evaluation methods are discussed in Section 2.3. 

### 2.1. The TDOA Localization Problem 

The emitter E is located at an unknown position $P_{0}=\left(x_{0},y_{0},z_{0}\right)$ and it emits a signal at an unknown time $T_{0}$. Sensors $S_{i},i=1,2,\ldots ,N$ are deployed at known positions $p_{i}=\left(x_{i},y_{i},z_{i}\right)$. The emitted signal is detected by sensor $S_{i}$ at time instant $t_{i},\;i=1,\;2,\;\ldots ,\;N$. If the propagation speed of the signal is c and there is line of sight (LOS) between E and $S_{i}$, then the detection time $t_{i}$ can be expressed as follows:

(1) $$t_{i}=T_{0}+\frac{\left|P_{0}-p_{i}\right|}{c}.$$

**Figure 1:**
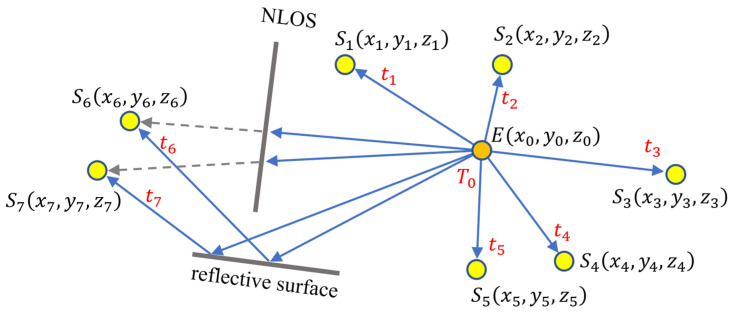
TDOA localization. Emitter E emits an event at unknown time instant $T_{0}$ at unknown location $\left(x_{0},\;y_{0},\;z_{0}\right)$. Sensor $S_{i}$ measures the time of detection $t_{i}$. From the measured $t_{i}$ and the known sensor positions $\left(x_{i},\;y_{i},\;z_{i}\right)$, the emitter position is estimated. Sensors $S_{6}$ and $S_{7}$ measure outliers, due to non-line-of-sight situations.

A measurement scenario is illustrated in Figure 1, where sensors $S_{1},\;S_{2},\;\ldots ,S_{5}$ are in LOS of the source, while $S_{6}$ and $S_{7}$ are in NLOS positions and provide measurements through a reflective surface. In the example, measured times $t_{1},t_{2},\ldots ,t_{5}$ satisfy (1), but for $S_{6}$ and $S_{7}$, the measured time is larger than the ideal time calculated from (1). 

The objective is to determine the position $P_{0}$ using the measurements $t_{i}$ and positions $p_{i}$. The task becomes more challenging when measurements contain outliers, such as those caused by NLOS conditions. 

Several solutions have been proposed to solve the TDOA problem. Closed-form solutions are available for a limited number of sensors [27,28,29,30,31,32], while iterative techniques are used for general cases [33,34]. Particle filters [35,43], Hough transform-based solutions [35,36,37], and consensus functions [38,39,40] have also been proposed. 

In most TDOA localization problems, the propagation speed of the signal is assumed to be known. In [44], a joint estimation method is proposed, which is capable of estimating both the source location and the propagation speed. The placement of anchors has a significant effect on the localization accuracy. The optimal geometry, considering the communication constraints that are important in practice, has been analyzed in [45].

Most solutions assume that measurements are correct and do not include outliers. In practical cases, however, outliers are common, due to NLOS situations, reflections, sensor faults, etc. Therefore, in real-world scenarios, the presence of outliers can severely bias location estimates unless they are removed from the estimation [41,42]. 

Several works address the NLOS problem. In [46], an iterative solution, based on the maximum correntropy criterion with a variable center, was proposed. Various methods using convex optimization and semidefinite programming have also been proposed [47,48,49]. However, these methods are computationally intensive. In [50], a neurodynamic optimization approach is proposed, which provides a trade-off between accuracy and computational complexity. 

Other approaches for providing accurate location estimates in the presence of outliers involve sensor consensus. RANSAC-based [51,52] methods randomly select a core group including a small number of sensors (typically 4–5) and use it to perform localization. The measurements from the other sensors are then checked against the core estimate, and the location estimate is refined. In an iterative process, several core groups are selected, and the best result is chosen. By conducting a high number of trials, the results are statistically correct with a high probability [53]. 

Hough transform-based solutions utilize a voting system in which each pair of sensors votes for a set of possible location estimates. The position with the highest number of votes becomes the location estimate. Fast calculation methods exist when the unknown location is in a plane [37].

Consensus functions also use a voting system, but unlike Hough transform and RANSAC, which use groups of a small number of sensors in the voting process, here, all of the sensors cast a single cooperative vote. The method was successfully utilized in three dimensional acoustic systems with a high number of unreliable measurements [38,39]. The detailed operation is reviewed in Section 2.2.

### 2.2. Consensus-Function-Based Localization

In this section, consensus functions are reviewed based on [38,39]. The distance $d_{i}$ between sensor $S_{i}$ and an arbitrary point $P=\left(x,y,z\right)$ is

(2) $$d_{i}\left(P\right)=d_{i}\left(x,y,z\right)=\sqrt{{\left(x_{i}-x\right)}^{2}+{\left(y_{i}-y\right)}^{2}+{\left(z_{i}-z\right)}^{2}}.$$

If the source position is $\left(x,y,z\right)$, then the emission time $T_{0}$ can be estimated from measurement $t_{i}$ using (1):

(3) $$T_{i}\left(P\right)=T_{i}\left(x,y,z\right)=t_{i}-\frac{d_{i}\left(x,y,z\right)}{c}.$$

Thus, $T_{i}\left(x,y,z\right)$ is the estimate of the emission time by a single sensor $S_{i}$, provided the source is at location $\left(x,y,z\right)$. Using all sensor measurements, the set of $T_{i}\left(x,y,z\right)$ $\left(i=1,2,\ldots ,N\right)$ estimates are available for an arbitrary point $\left(x,y,z\right)$. From the set of $T_{i}\left(x,y,z\right)$, the consensus group is formed, according to Definition 1.

**Definition** **1.** *A consensus group at position* $\left(x,y,z\right)$ *with window length* w *contains an S subset of* $T_{i}\left(x,y,z\right),$ *such that for any two elements* $T_{j}\left(x,y,z\right),\;T_{k}\left(x,y,z\right)\in S,$ *it is true that* $\left|T_{j}\left(x,y,z\right)-T_{k}\left(x,y,z\right)\right|<w.\;$

In the ideal case when there are no measurement errors present, for the true location $\left(x_{0},y_{0},z_{0}\right)$, all $T_{i}\left(x_{0},y_{0},z_{0}\right)$ estimates are equal to the true emission time $T_{0}$. In real cases when measurement noise is present, the $T_{i}$ estimates will be in the close vicinity of $T_{0}$, forming a large consensus group. The extent of the ‘vicinity’ is dependent on the level of noise and characterized by the window length w. Sensors that produce outlier measurements may have an estimated value $T_{i}$ that is significantly different from $T_{0}$, and these estimates will not be members of the consensus group. 

**Figure 2:**
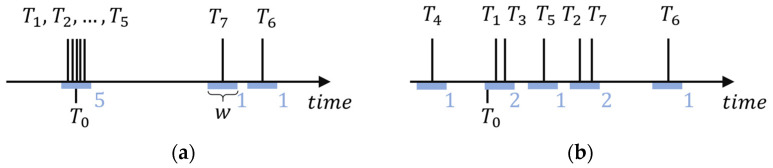
The calculation of the consensus function, where measurements $t_{1},\;t_{2},\;\ldots ,\;t_{5}$ are correct with small measurement noise, while $t_{6}$ and $t_{7}$ are outliers. (**a**) Estimated emission times $T_{1},\;T_{2},\ldots ,T_{7}$ at the true source position. There is a consensus group of five emission time estimates around $T_{0}$; the value of the consensus function is 5. (**b**) Estimated emission times at another position. The largest consensus group contains only two estimates; the value of the consensus function is 2. Blue numbers indicate the cardinalities of the consensus groups.

An illustration is shown in Figure 2a, where estimates $T_{1},T_{2},\ldots ,T_{5}$ form a five-element consensus group around time $T_{0}$. Estimates $T_{6}$ and $T_{7}$, corresponding to outlier measurements, are not part of the consensus group (they form one-element consensus groups).

**Definition** **2.** *The value of the consensus function at point $\left(x,y,z\right)$* *is defined as the cardinality of the largest consensus group for location $\left(x,y,z\right)$.*

In the example of Figure 2a, the value of the consensus function is 5. For locations $\left(x,y,z\right)$ other than $\left(x_{0},y_{0},z_{0}\right)$, estimates $T_{i}\left(x,y,z\right)$ are likely to differ significantly, resulting in the formation of small consensus groups rather than one large group. Figure 2b shows an example where the consensus function is evaluated at a point other than the true source location. The estimates $T_{i}$ are scattered and the largest consensus groups contain only two sensors; thus, the value of the consensus function is 2.

Note that sensors corresponding to a consensus group calculated at point $\left(x,y,z\right)$ agree on the hypothesis that the source is located at position $\left(x,y,z\right)$. The more sensors in consensus, i.e., the higher the consensus function, the higher the likelihood that the source is actually at position $\left(x,y,z\right)$. Therefore, the estimated source location is the one with the highest consensus function.

To create consensus groups, the maximum difference w allowed between group members is defined. In Figure 2, a sliding window with width w is shown. The window function $\Lambda_{w}$ of width w is defined as follows:

(4) $$\Lambda_{w}\left(t\right)=\left\{\begin{array}{ll} 1 & \mathrm{if}\;\left|t\right|<\frac{w}{2}\\ 0 & \mathrm{otherwise} \end{array}\right.$$

Using the window function, the consensus function can be expressed as follows:

(5) $$C_{w}\left(x,y,z\right)=\underset{t\in \mathbb{R}}{\max}\overset{N}{\underset{i=1}{\sum }}\Lambda_{w}\left(T_{i}\left(x,y,z\right)-t\right)$$

The set of points where the consensus function $C_{w}\left(x,y,z\right)$ takes its maximum is

(6) $$\Psi_{w}=\underset{\left(x,y,z\right)}{\mathrm{argmax}}\left(x,y,z\right).$$

The location estimate $\left({\hat{x}}_{0},{\hat{y}}_{0},{\hat{z}}_{0}\right)\;$is calculated as the mean of the positions in $\Psi_{w}$:

(7) $$\left({\hat{x}}_{0},{\hat{y}}_{0},{\hat{z}}_{0}\right)=\frac{1}{\left|\Psi_{w}\right|}\underset{p\in \Psi_{w}}{\sum }p.$$

The window width w is essential in calculating the consensus function as it determines the required proximity of estimated emission times $T_{i}$ within a consensus group. At the true location, the estimates $T_{i}$ should be treated as a single group, despite the small perturbations in the values caused by measurement noise, as shown in Figure 2a. This requirement leads to a lower bound on w, as follows. The accuracy of $T_{i}$ depends on the accuracy of measurements $t_{i}$ and the precision of the sensor locations $p_{i}$. If the maximum sensor location error is Δs and the maximum time measurement error is Δτ, then, from (3), the maximum error of $T_{i}$ is

(8) $$\Delta T_{max}=\Delta s/c+\Delta \tau .$$

Since the maximum difference between any two values of $T_{i}$ is $2\Delta T_{max}$, this is the lower bound on w. To keep w as small as possible, the window width is set to

(9) $$w=2\Delta T_{max}=2\left(\frac{\Delta s}{c}+\Delta \tau \right).$$

In practical scenarios, a rough a priori estimate of the measurement errors Δs and Δτ is required. From these estimates, the design parameter w can be determined using (9). 

### 2.3. Calculation of the Consensus-Function-Based Location Estimate

The source location is determined by finding the maximum of the consensus function. However, this can be challenging because the consensus function is not smooth, being an integer-valued function, and it may have several local maxima. Traditional gradient-based search methods do not work for such functions. An exhaustive search on a finite grid provides the global maximum; however, for large target areas, especially in 3D, it is not practical. To provide a faster solution, a fast heuristic calculation was proposed in [38,39]. This method is based on integer arithmetic and the Generalized Bisection method and searches for the source location and emission time in a four-dimensional space (x, y, z, t). The performance of the search method has been experimentally validated [38], but the correctness of the algorithm has not been proven. 

Another fast solution was proposed in [53] using a RANSAC-based approach. Random sets of five measurements were utilized to calculate the core estimate, which was then compared to the other measurements using the consensus function. This method is able to provide the correct estimates with high probability if the number of random trials is high enough [53]. 

A fast Branch and Bound-type calculation method has been proposed recently [40]. A global convergence property of the method has been reported, supported by an experimental validation. The following section presents an improved version of [40] and provides a mathematical proof of its global convergence property. 

## 3. Fast Consensus-Based Localization

The fast search method proposed in [40] performs the search on a grid for the maximum of the consensus function. The grid is iteratively refined until the required grid size is reached. During the refinement process, areas that are unlikely to contain the global maximum are excluded from a further search; thus, the search method converges quickly. The method’s correctness was validated through simulation experiments. Now, an enhanced and more detailed version of the algorithm [40] is proposed in Section 3.1. The correctness of the algorithm is proven in Section 3.2 and Section 3.3.

### 3.1. Fast Search on a Finite Grid

The proposed fast search method is described in Algorithm 1, followed by an illustration of its operation.**Algorithm 1** Branch and Bound algorithm to find the maximum of the consensus function **Input:**1.measurements: $t_{i},\;i=2,3,\ldots ,N\;$2.sensor positions: $\left(x_{i},y_{i},z_{i}\right),\;i=1,2,\ldots ,N$3.required (fine) grid size: Δ**Initialization:**4.Define a grid $G_{f}$ over the search area with a grid cell size equal to Δ. This is the fine grid where the maximum of the consensus function is searched for.5.Cover the search area with a coarse grid $G_{c}$ using grid cells of size $2^{k}\Delta ,$ where k is an integer greater than 1, so that the grid points of $G_{c}$ overlap with some of those of $G_{f}$. The search starts from this grid.6.Mark each cell of $G_{c}$ as active.7.Calculate the upper bound ${\bar{C}}_{w}$ of $C_{w}$ for each cell**Iteration: repeat Branch and Bound while active cells exist** **Branch:**8.Select the active cell with the highest ${\bar{C}}_{w}$ value and refer to it as Q. If multiple cells have the same highest value, select the one with the smallest size.9.Replace Q with smaller cells $Q_{i}^{\prime }$ that have a linear size half of the original (i=1,2,3,4 in 2D and i=1,2,…,8 in 3D).10.If $Q_{i}^{\prime }$ is completely outside of the search area, mark it passive.11.If the size of $Q_{i}^{\prime }$ is larger than Δ then mark $Q_{i}^{\prime }$ as active and recalculate the upper bound ${\bar{C}}_{w}$ for $Q_{i}^{\prime }$12.If the size of $Q_{i}^{\prime }$ is equal to Δ then mark $Q_{i}^{\prime }$ as final and calculate $C_{w}$ in the center of the cell**Bound:**13.Calculate the maximum$\;C_{max}$ of the $C_{w}$ values in the final cells.14.Mark active cells with ${\bar{C}}_{w}<C_{max}$ as passive**Output:**15.Collect the final cells with $C_{w}=C_{max}$ to form $\Psi_{w}$$.\;\mathrm{Then},\;\mathrm{calculate}\;\left({\hat{x}}_{0},{\hat{y}}_{0},{\hat{z}}_{0}\right).$

The algorithm finds the maximum of the consensus function using a finite grid with a grid size of Δ (step 3), i.e., in 2D, the grid cells are squares of size Δ×Δ and in 3D, the cells are cubes of size Δ×Δ×Δ. The consensus function is evaluated in the center of the cells. Note that the grid size determines the resolution of the search. 

**Figure 3:**
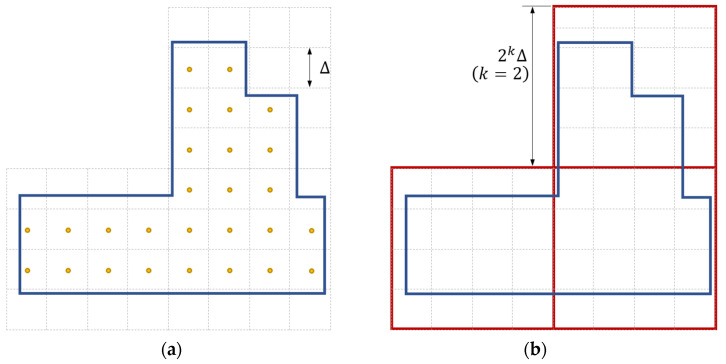
(**a**) The search area is surrounded by a solid blue line, and the fine search grid is represented by dashed grey lines. The consensus function is to be evaluated at the center points of the search grid, marked by orange dots. (**b**) The coarse grid, which is represented by the red lines, is placed over the fine grid and used as the initial grid.

Figure 3 illustrates the operation, using a 2D example. Figure 3a shows the fine grid over the search space (step 4). Then, a coarse grid (in red) is placed over the fine grid (step 5). The size of the coarse grid must be a $2^{k}$ multiple of Δ; in the example k=2, the coarse cells are thus of size 4Δ×4Δ, as shown in Figure 3b. Note that in practical cases, k is typically much larger than the small value used in this example.

**Figure 4:**
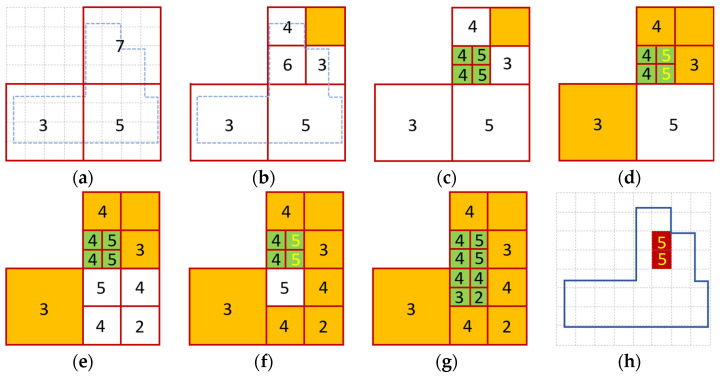
Branch and Bound search to find the maximum of the consensus function. Active, passive, and final cells are denoted by white, orange, and green colors, respectively. Upper bounds are shown in the active and passive cells, and the actual consensus function values are shown in the final cells. The highest consensus values in each iteration are shown by yellow numbers. (**a**) The fine grid (dashed grey lines) and the coarse initial grid (solid red lines), with the initial upper bounds. The search area is also shown by dashed blue lines. (**b**) The branch step for the cell with an upper bound of 7. The cell outside of the search region is set to passive. The bound step is idle. (**c**) The branch step for the cell with an upper bound of 6. The final cell size is reached for the new cells. (**d**) The bound step. The highest consensus value is 5 (shown by yellow numbers); active cells with an upper bound less than 5 are set to passive (yellow rectangles). (**e**) The branch step for the active cell with an upper bound of 5. (**f**) The bound step. The highest consensus value is 5; active cells with a smaller upper bound are set to passive. (**g**) The branch step for the active cell with an upper bound of 5. There are no more active cells. (**h**) The cells with the highest consensus value, indicated by a red color, comprise the set of $\Psi_{w}$.

The further operation is illustrated in Figure 4. In Figure 4a, the three starting cells are active, indicated by the white background, and the upper bounds ${\bar{C}}_{w}$ are displayed within each cell (according to steps 6–7). Figure 4b shows the first branching step. The highest upper bound is seven, so the corresponding cell is selected and divided into four smaller cells (steps 8–9). One cell is outside of the search region and is marked as passive (step 10), shown by an orange background. The new cell size is still larger than Δ, so the upper bounds are calculated for the new cells (step 11). As there are no exact consensus values calculated yet, the bound step is idle. Figure 4c illustrates the subsequent branching step, where the cell with an upper bound of 6 is divided into four smaller cells. The new cell size is now Δ, so the new cells are marked as final (shown by green background) and the exact consensus values are calculated in these cells (step 12). The next bound step is shown in Figure 4d. The value $C_{max}$ is 5 (step 13), and the maximum values are shown by a yellow color in the corresponding cells. Several cells with ${\bar{C}}_{w}<5$ are marked as passive (step 14), shown by an orange background color. Figure 4e shows the next branching step of the active cell with an upper bound of 5. The subsequent bound step is shown in Figure 4f: $C_{max}$ is 5, and thus the cells with upper bound values below 5 are marked as passive. In Figure 4g, the next branch step is shown, where the cell with ${\bar{C}}_{w}=5$ is divided into four final cells. Since there are no more active cells, the bound step is idle and the iteration is finished. Figure 4h shows the cells with $C_{w}=C_{max}=5$, forming $\Psi_{w}$ (step 15).

The design parameter Δ defines the required resolution and thus accuracy, since the search method finds the maximum of the consensus function of the defined grid. Small Δ results in a finer resolution but potentially requires more computation. It is therefore inadvisable to select values for Δ that are excessively small.

In practical situations, the size of Δ can be determined along with parameter w, as follows: The window width w is determined from the time and distance measurement errors, according to (9). The window width w reflects the measurement errors in terms of timing error, while the quantity c·w quantifies the measurement errors in terms of distance. Although the overall positioning accuracy that can be achieved is dependent on the placement of the sensors [44], the order of magnitude can be estimated by c·w. Thus, a practically good design choice for grid size Δ lies in the range of 0.1c·w…c·w.

### 3.2. Upper Bound of the Consensus Function

In the operation of Algorithm 1, the upper bound ${\bar{C}}_{w}$ of the consensus function in a cell is essential. In [40], a quantity was proposed for ${\bar{C}}_{w}$ and it was validated through simulations. However, to date, no proof has been found to support that the proposed quantity is indeed an upper bound. In the following, a mathematical proof will be provided.

The following theorem, Theorem 1, provides an upper bound for the consensus function in a spherical region.

**Theorem** **1.** *Let point P* *be the center of a sphere S* *with radius r* *and let Q* *be a point inside S* *. If $w^{\prime }=w+\frac{2r}{c}$, then the following inequality holds:*

(10) $$\underset{Q}{\max}C_{w}\left(Q\right)\leq C_{w^{\prime }}\left(P\right).$$

**Figure 5:**
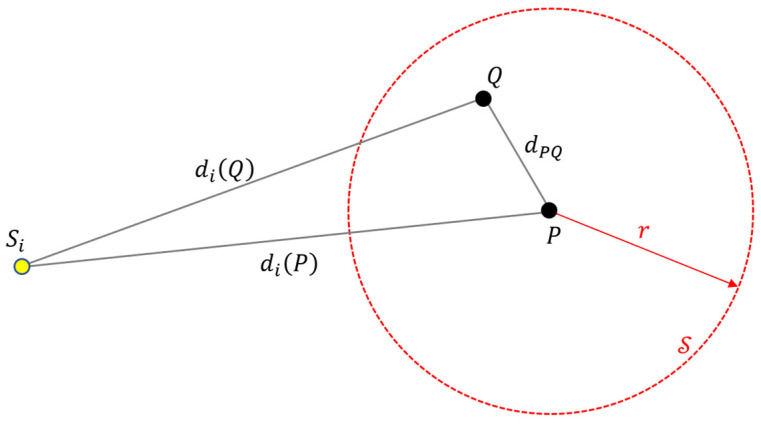
Geometry of points P, Q, and sensor $S_{i}$ in the proof of Theorem 1.

**Proof of Theorem** **1.** Points P, Q, and sensor $S_{i}$ are shown in Figure 5, along with the sphere S around P. The distance between points P and Q is denoted as $d_{PQ}$. Let us define the quantity $\Delta d_{i}$ as follows:

(11) $$\Delta d_{i}=d_{i}\left(P\right)-d_{i}\left(Q\right).$$

Since Q is inside S, trivially

(12) $$d_{PQ}\leq r.$$

By applying the triangle inequality to the triangle $PQS_{i}$ of Figure 5, it also follows that

(13) $$d_{PQ}\geq d_{i}\left(P\right)-d_{i}\left(Q\right).$$

By combining (11)–(13), it follows that

(14) $$\Delta d_{i}\leq r.$$

Let the value of the consensus function $C_{w}$ at point Q be denoted by $K=C_{w}\left(P\right)$. Thus, according to (5) and (4), there exists a time instant $t_{0}$ and a set Θ of K sensors for which the following holds:

(15) $$\left|T_{i}\left(P\right)-t_{o}\right|<\frac{w}{2},\;S_{i}\in \Theta .$$

For the sake of simplicity, but without loss of generality, let us assume that the sensors in Θ are $S_{1},\;S_{2},\;\ldots ,\;S_{K}$. In the proof, index k refers to these sensors, specifically k=1, 2, …, K.

Using (3), the inequality (15) can be written as

(16) $$\left|t_{k}-\frac{d_{k}\left(Q\right)}{c}-t_{o}\right|<\frac{w}{2}.$$

Using (11), the following holds for point P:

(17) $$\left|t_{k}-\frac{d_{k}\left(P\right)}{c}-t_{o}\right|=\left|t_{k}-\frac{\Delta d_{k}+d_{k}\left(Q\right)}{c}-t_{o}\right|=\left|t_{k}-\frac{d_{k}\left(Q\right)}{c}-t_{o}-\frac{\Delta d_{k}}{c}\right|.$$

By utilizing the basic inequality $\left|a-b\right|<\left|a\right|+\left|b\right|,$ from (17), it follows that

(18) $$\left|t_{k}-\frac{d_{k}\left(P\right)}{c}-t_{o}\right|\leq \left|t_{k}-\frac{d_{k}\left(Q\right)}{c}-t_{o}\right|+\left|\frac{\Delta d_{k}}{c}\right|.$$

Applying (14) and (16), inequality (18) leads to

(19) $$\left|t_{k}-\frac{d_{k}\left(P\right)}{c}-t_{o}\right|\leq \frac{w}{2}+\frac{r}{c}$$

and thus using (3), it follows that

(20) $$\left|T_{k}\left(P\right)-t_{o}\right|\leq \frac{w}{2}+\frac{r}{c}=\frac{1}{2}\left(w+\frac{2r}{c}\right)=\frac{w^{\prime }}{2}.$$

Comparing (4) and (20) leads to

(21) $$\Lambda_{w^{\prime }}\left(T_{k}\left(P\right)-t_{o}\right)=1.$$

Note that (21) holds for all K sensors $S_{1},S_{2},\ldots ,S_{K}$. Therefore, using (5) and (21), it follows that

(22) $$C_{w^{\prime }}\left(P\right)=\underset{t\in \mathbb{R}}{\max}\overset{N}{\underset{i=1}{\sum }}\Lambda_{w^{\prime }}\left(T_{i}\left(P\right)-t\right)\geq \overset{K}{\underset{k=1}{\sum }}\Lambda_{w^{\prime }}\left(T_{k}\left(P\right)-t_{0}\right)=\overset{K}{\underset{k=1}{\sum }}1=K=C_{w}\left(Q\right).\;$$

Thus, for any point Q inside S, $C_{w^{\prime }}\left(P\right)\geq C_{w}\left(Q\right)$, which leads directly to (10). □

Using the results of Theorem 1, Theorem 2 provides an upper bound for the consensus function in a cell.

**Theorem** **2.** *Let us denote the dimension of the search space with D. Let point $P_{C}$* *be the center of a cell C* *of size L, where C* *is a cube for D=3* *or a square for D=2. Then, for all points Q* *inside C,*

(23) $$\underset{Q}{\max}C_{w}\left(Q\right)\leq C_{w_{L}}\left(P\right)$$

*with*

(24) $$w_{L}=w+\frac{L\sqrt{D}}{c}.$$

**Proof of Theorem** **2.** For a cube of size L, the maximum distance between its center $P_{C}$ and any point Q inside the cube is $\frac{L\sqrt{3}}{2}$. For a square of size L, the maximum distance between $P_{C}$ and any point Q inside the square is $\frac{L\sqrt{2}}{2}$. Thus, for a cell of dimension D (D=2,3), the cell is inside of a sphere with center $P_{C}$ and radius $r=\frac{L\sqrt{D}}{2}$. With the choice of

(25) $$w^{\prime }=w+\frac{2r}{c}=w+\frac{L\sqrt{D}}{c}=w_{L},$$

Theorem 1 yields (23). □

### 3.3. Global Convergence

Finally, the global convergence property of Algorithm 1 is proven.

**Theorem** **3.** *If the upper bound ${\bar{C}}_{w}$* *for a cell with center P* *and size L* *is computed as ${\bar{C}}_{w}=C_{w_{L}}\left(P\right)$* *with $w_{L}=w+\frac{L\sqrt{D}}{c}$, then Algorithm 1 finds the global maximum of $C_{w}$* *on the finite grid $G_{f}$.*

**Proof of Theorem** **3.** Let $C_{MAX}$ be the maximum of the consensus function inside the search area, calculated over $G_{f}$, and let $C^{*}$ denote a particular cell of $G_{f}$ inside the search area, where the consensus function is $C_{MAX}$. In Algorithm 1, the initial cells are iteratively divided into smaller cells in the branching steps until the required size of Δ is reached, the cells marked as final being a subset of the cells of $G_{f}$. It will be shown that at the end of Algorithm 1, $C^{*}$ is in the set of final cells.

In Algorithm 1, the consensus values are calculated in the final cells (step 12), which are the cells of the fine grid $G_{f}$. Therefore, for the value $C_{max}$ calculated in step 13, it is true that $C_{max}\leq C_{MAX}$. Note also that for any cell of size L that contains $C^{*}$, according to Theorem 2, ${\bar{C}}_{w}\geq$ $C_{MAX}$. Therefore, in the bounding phase (steps 13–14) for a cell containing $C^{*}$, ${\bar{C}}_{w}\geq C_{max}$, so this cell cannot be marked passive in step (14). The same cell cannot be made passive in step 10 either, because $C^{*}$ is located inside the search area.

At the beginning of the algorithm, $C^{*}$ is part of a larger active cell (step 6). Active cells are eventually (a) divided into smaller active cells (steps 8–9) or (b) smaller final cells (step 12) or (c) marked as passive (steps 10 and 14). In case (a), $C^{*}$ is preserved in one of the active cells, while in case (b), $C^{*}$ becomes one of the final cells. Case (c) does not apply to a cell containing $C^{*}$, as was shown above. Thus, a cell containing $C^{*}$ is either active or final. Since the algorithm always divides the active cells into smaller cells, at the end of the iteration, the cell containing $C^{*}$ necessarily becomes one of the final cells.

It has been shown that every cell of $G_{f}$ that gives the maximum consensus function is part of the set of final cells. Since the maximum is searched in this set (step 15), Algorithm 1 finds all of the global maxima over the grid $G_{f}$. □

## 4. Performance Evaluation

The performance of the proposed consensus-function-based method (CF) is evaluated by simulation examples and real measurements. For comparison, we use the iterative LS algorithm and the three-dimensional extension of the COM-W algorithm [32], which provides a closed-form solution. 

### 4.1. Simulations

**Figure 6:**
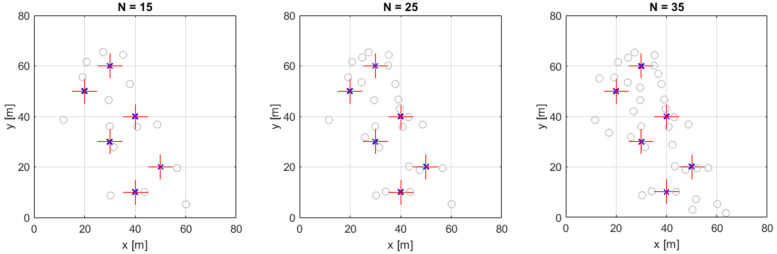
The 3D simulation experiment with additive measurement noise and no outliers. Sensor positions are denoted by grey circles, and red crosses indicate the target positions. Overlapping magenta, cyan, and blue x’s show the estimated target positions of LS, COM-W, and CF, respectively.

The simulations utilize a sensor setup that was previously used in a real shooter localization experiment [38]. The bird’s eye views of the simulation setups are shown in Figure 6, using 15, 25, and 35 sensors, denoted by grey circles. The elevations of the sensors were in the range of −0.4 m to 10.9 m. The six simulated target positions are shown by red crosses and are listed in Table 1. As Table 1 shows, there were 2D and 3D experiments. In the 2D experiment, the target elevation was known to be at 0 m. This situation is common when the target, such as an autonomous vehicle, is moving on a plane. In the 3D experiments, the target elevation was also to be determined, which is a typical scenario when trying to locate a flying object. It is important to note that the sensor positions were three-dimensional in both the 2D and 3D experiments.

The simulations were carried out as follows:

- For each target position, we calculated the exact distances $d_{i}$ between the target position and the sensors.
- We calculated the exact times of arrivals $t_{i}$ as $d_{i}/c$.
- We added measurement noise with normal distribution $N\left(0,\sigma \right)$ to $t_{i}$ and added additional measurement noise with normal distribution $N\left(0,\;100\sigma \right)$ to $t_{i}$ to emulate the faulty (outlier) sensors. The number of outliers was $N_{out}.\;$For each target position, 100 independent measurements were created.
- Using measurements $t_{i}$ and the sensor positions $p_{i}$, the estimated target positions were calculated by LS, COM-W, and CF.
- The tests were conducted using Matlab version R2021b on a computer with i5-8265 CPU with clock frequency of 1.6 GHz, and 24 GB of RAM.
- The LS algorithm was started from a random position within 1 m of the true position.
- The CF method was implemented in Matlab according to Algorithm 1. Apart from Matlab’s built-in vector operations, no acceleration methods (e.g., multithreading) were used.
- The final resolution of the CF method was Δ=0.1 m.

In the first experiment, there were no outliers present and σ was 0.3 ms (equivalent to a distant measurement error of 0.1 m). The estimated positions for the 3D case are shown in Figure 6 with markers ‘x’, close to the true positions. Table 2 and Table 3 summarize the results for the 2D and 3D experiment, respectively: for each target position, the square root of the Cramer–Rao lower bound (CRLB) is shown, followed by the root-mean-square error (RMSE) of the tested algorithms. In this test, all three methods performed very well with an RMSE close to the theoretical optimum ($\sqrt{CRLB}$). According to the results, there is no practical difference in accuracy among the three methods in this outlier-free experiment.

**Figure 7:**
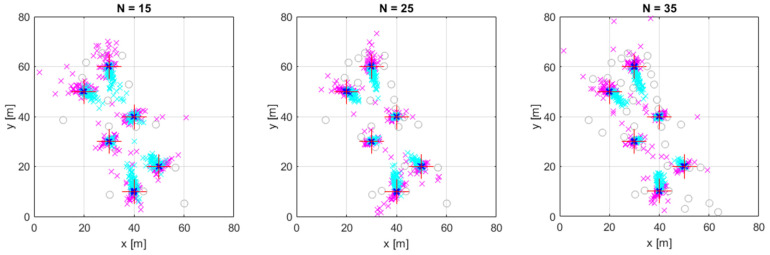
The 3D simulation experiment with additive measurement noise and outliers. Sensor positions are denoted by grey circles, and red crosses indicate the target positions. Magenta, cyan, and blue x’s show the estimated target positions of LS, COM-W, and CF, respectively.

In the second experiment, we created outlier measurements as well. We selected the number $N_{out}$ of outliers randomly among $1\leq N_{out}\leq 6$, independently in each experiment. In each experiment, we generated $N-N_{out}$ correct measurements with a noise level of σ and $N_{out}\;$outliers with a noise level of 100σ. The results for the 3D case are shown in Figure 7. Comparing Figure 6 and Figure 7, it is apparent that the LS and COM-W methods have significantly increased variance, with errors occasionally reaching tens of meters. The CF method, however, has a small estimation error. The detailed results for the 2D and 3D cases are listed in Table 4 and Table 5, respectively. Clearly, neither the LS nor the COM-W tolerated well the presence of outliers; both algorithms had several meters of errors (the LS solver also diverged several times, but these cases are omitted from the analysis). In 2D, the mean error of LS and COMW methods increased to 2–3 m, while in 3D, the error of these methods was between 3 and 8 m. The CF method, however, provided low error levels, in 2D, around 0.2 m and in 3D, between 0.3 m and 0.5 m, which was close to the theoretical optimum. 

**Figure 8:**
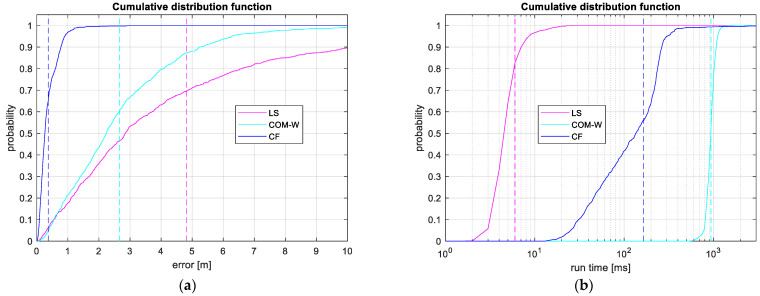
The cumulative distribution function of the localization error in 3D (**a**) and the execution time (**b**) at position #1, using 25 sensors and the maximum 4 outliers. The dashed lines show the mean error values and mean execution times.

The distribution of the error is somewhat visible in Figure 7, but for a more comprehensive visualization, Figure 8a presents the cumulative distribution function (cdf) of the localization error for the LS, COM-W, and CF methods. To create the figure, we conducted 1000 simulation experiments for position #1 in 3D, with $N=25,\;1\leq N_{out}\leq 4$. The vertical dashed lines show the mean error values, corresponding to the values shown in Table 5.

The comparison of the results of the CF method in the first and second experiments shows that the level of RMSE increases when outliers are present. Note that the theoretical limit (CRLB) corresponds to the case where every sensor provides good measurements, which is not the case here. The reduction in the number of functional sensors led to an increase in the error level.

We measured the mean execution times of the algorithms as a function of the number of sensors and they are presented in Table 6. In the experiments, the LS method was the fastest, with an execution time of approximately 4 ms in the 2D case and 5 ms in the 3D case. The CF method required approximately 20 ms in 2D and 90 ms in 3D. The slowest method was COM-W with an average execution time of more than 200 ms in 2D and 1.3 s in 3D. It is also apparent that the execution time of the COM-W method strongly depends on N, whereas the LS and CF methods did not show significant dependence on it. As an example, the cumulative distribution functions of the execution times for the 3D experiment at position #1 are shown in Figure 8b. The vertical dashed lines show the mean execution times for this particular experiment. The sharp transition of COM-W indicates that the execution time of COM-W is quite deterministic, with an average of approximately 950 ms. The iterative LS has a run-time that varies between 3 and 20 ms, with a mean value of 6 ms. The execution time of CF is also dependent on the specific scenario, with the majority of values falling between 10 and 400 ms, with a mean value of 165 ms.

**Figure 9:**
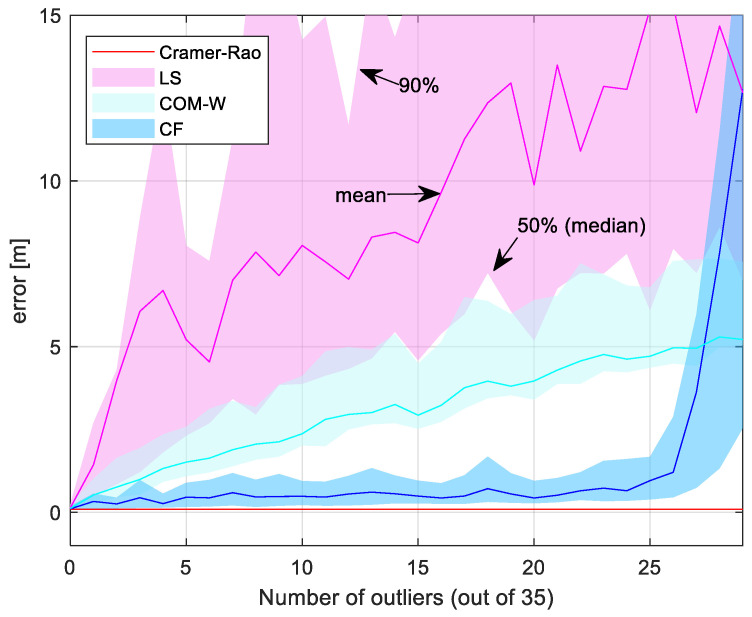
Fault tolerance test results. Solid lines show the mean error, and the shaded regions indicate the error between the 50th and 90th percentiles.

The experiment in Figure 9 illustrates the fault tolerance of CF. In the test, we used target position #5 with 35 sensors, and varied the number of outliers from 0 to 25, with the outlier sensors chosen randomly. For each outlier number, we conducted 100 independent experiments, and measured the RMSE for the methods CF, LS, and COM-W. The figure shows the mean estimation errors with solid lines, and the upper and lower edges of the shaded areas correspond to the 90th percentile and the 50th percentile (median) of the error, respectively. 

As Figure 9 shows, the error levels for all three methods are close to the Cramer–Rao lower bound, when there are no outliers present. When the first outliers appear, the error of LS and COM-W increases to several meters and the error continues to increase for a higher number of outliers. It is noteworthy that the COM-W method tolerates outliers better than the LS method. The error of the CF method increased slightly with the presence of outliers but remained close to the theoretical value until the number of bad measurements exceeded 25 (i.e., 71% of the total number of 35). Above this outlier number, the performance of the CF method decreased significantly. The ability of the CF method to tolerate more than 70% of sensors being outliers clearly demonstrates its robustness.

### 4.2. Measurements

**Figure 10:**
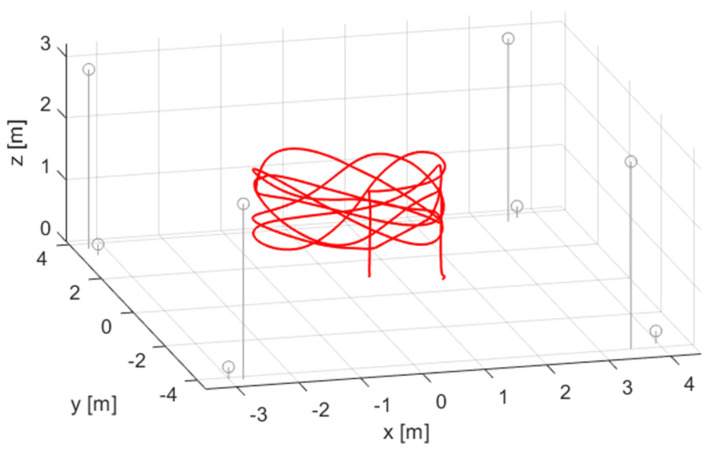
Sensor placement and trajectory of the indoor experiment. Grey circles indicate the sensor positions, and the red curve shows the trajectory of the flying target.

The performance of the CF method was evaluated using two real-world measurements. The first measurement was obtained from the public database UTIL [54], where measurements were performed using ultrawideband (UWB) radios. The data were collected in an indoor flight arena of size 7 m × 8 m × 3.5 m. The setup comprised 8 fixed UWB anchors, and the target was a UWB unit mounted on a flying quadrotor platform. The reference trajectory of the target was measured by a millimeter-accuracy optical system, using 10 Vicon Vantage+ cameras. We used measurement record const4-trial6-tdoa2-traj3, which contained obstacles (wooden and metal boxes) to create challenging NLOS measurements [54]. The measurement setup is shown in Figure 10, where the grey circles indicate the beacons and the red line represents the target trajectory. 

**Figure 11:**
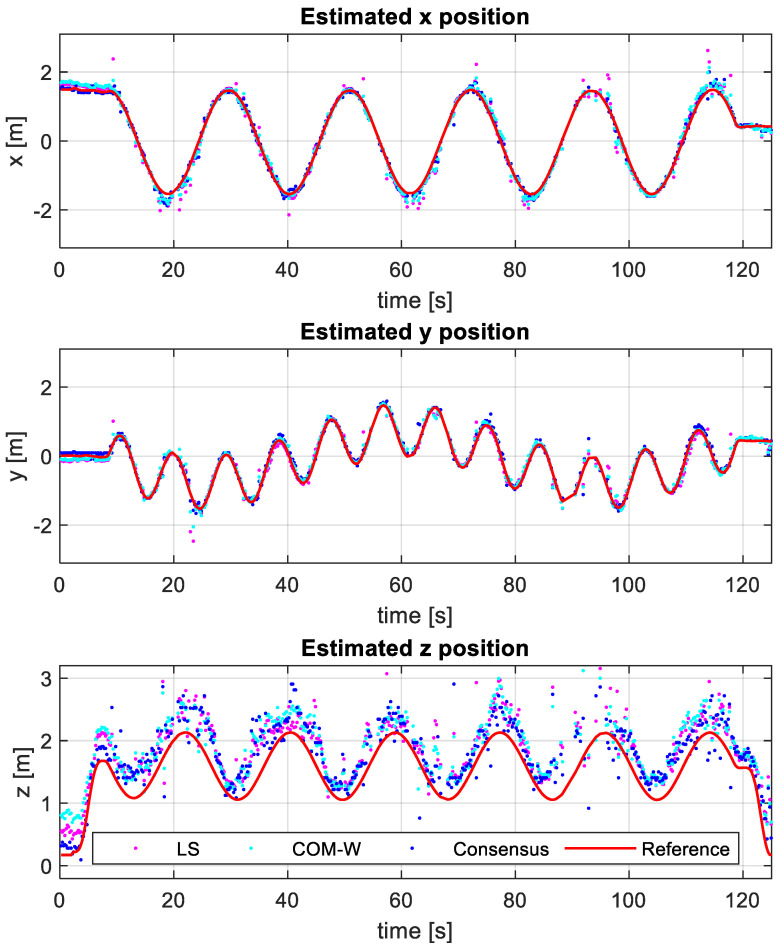
Estimated target positions of the indoor experiment.

Note that in the tests, we estimated the target position in every measurement position separately, and no filters (e.g., Kalman filters) were utilized to enhance the estimation quality. The estimated target positions are shown as colored dots in Figure 11, for the LS, COM-W, and CF methods. According to the results, the vertical inaccuracy is significantly higher than the inaccuracy in either the x or y direction. This is due to the particular setup of the beacons. The horizontal-only (RMSE-xy) and three-dimensional (RMSE-xyz) values are shown in Table 7. The 2D errors are around 0.2 m, while the 3D error is around 0.5 m. In this experiment, the LS method had the highest RMSE of 0.62 m, while the COM-W handled the NLOS situations better with an RMSE of 0.47 m. The CF provided the most accurate estimates with an error of 0.41 m. Note that the accuracy of the reference algorithm in [54] is 0.45 m, which was also outperformed by CF. 

The execution times are also shown in Table 7. In this setup, both LS and COM-W required approximately 4 ms to provide an estimate, while the execution time of CF was around 11 ms. 

**Figure 12:**
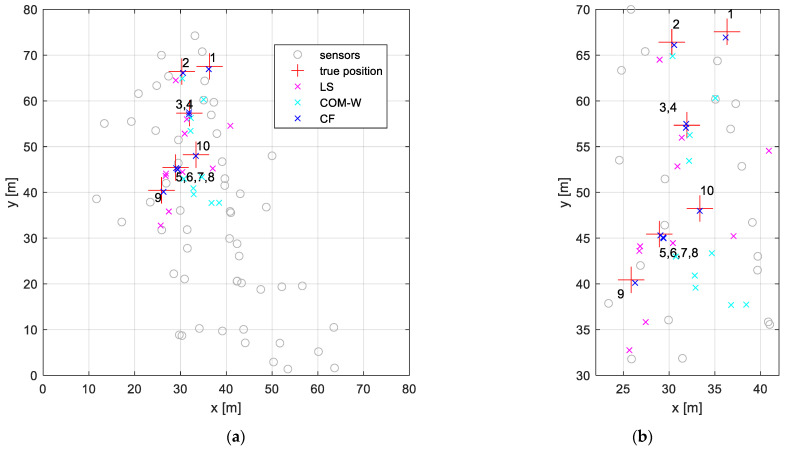
Bird’s eye view of the shooter localization experiment. The sensor elevations were in the range of −0.4 m to 10.9 m. (**a**) Full field and (**b**) enlarged view around the target positions.

The data of the second measurement are from a counter-sniper experiment [38]. The experiment was conducted in a village that had been utilized for military training. The village comprised several streets and numerous buildings. The 57 acoustic sensors were deployed on the street level and the window sills. The shooter fired a sniper weapon from various reference positions and the muzzle blast of the weapon was detected by the sensors. The ten reference shooter positions are listed in Table 8. The sensors are represented by grey circles in the bird’s eye view of Figure 12, where the elevations were between −0.4 m and 10.9 m. Note that the setup is a challenging NLOS situation: in each experiment, multiple sensors lacked a direct line of sight. These sensors were either unable to detect the muzzle blast, or they detected signals that were reflected by one or more of the walls of the buildings. The reflected signals arrived significantly later than the signals that should have arrived in a line-of-sight scenario (see Figure 1), so these measurements must be considered outliers.

In Figure 12, the estimated positions are shown by magenta, cyan, and blue x markers for the LS, COM-W, and CF, respectively. The enlarged image illustrates that the CF method produced estimations that closely match the true positions, whereas the LS and COM-W methods had significantly larger errors. The corresponding estimation errors are listed in Table 9, along with the number N of sensors providing measurement results and the consensus function value $C_{w}$. In every case, there is a difference between N and $C_{w}$, which means measurements with large errors are present. The significant errors are due to the lack of line of sight for a number of the sensors. The outliers cause high estimation errors for LS and COM-W. The mean error was 6.6 m for COM-W and 6.8 m for LS. The maximum error was 12.5 m for COM-W and 14.2 m for LS. The CF method tolerated outliers much better: the mean error was below 0.9 m, while the maximum error was 1.9 m. 

The execution times of the algorithms are also shown in Table 9. The LS method was the fastest, with a mean run-time of 18 ms. The CF required 153 ms on average, while for COM-W, the mean execution time was 949 ms. Note that the execution time of COM-W depends on the number *N* of sensors, while for LS and CF methods, the speed of convergence depends on the actual shape of the error surface, which is hard to predict. 

In the case of the indoor measurement, the CF method demonstrated comparable speed with the LS and COM-W methods. In the context of the shooter problem, however, the CF method was significantly slower than the LS method, yet proved to be considerably faster than the COM-W method. The indoor measurement is a typical small-scale problem, whereas the shooter application can be considered a large-scale problem. According to experiments, the CF scales with the number of sensors reasonably well, and the method can be applied for large-scale problems. 

The results of the run-time measurements indicate that the execution time of the LS method is only slightly affected by the actual measurement setup, and that it is not directly dependent on the number of sensors. The execution time of the COM-W is influenced by the number of sensors only. The run-time of the CF is in part dependent on the number of sensors and the actual shape of the consensus function. Consequently, the speed of the CF method is the least predictable of the three methods, especially in dynamically changing environments.

The shooter test database allows for a comparison of the proposed CF and previous consensus-based methods. In [38], the Generalized Bisection method was applied to accelerate the computation, while in [53], a mixed RANSAC–consensus approach was utilized. The brute-force computation of the consensus function in the 80 m×80 m×5 m search space with a grid size of Δ = 0.1 m would require a $3.2\times 10^{7}$ evaluation of the consensus function. The Generalized Bisection method was reported to use $10^{5}$ consensus function calls [38], while the RANSAC–consensus solution required $1.8\times 10^{4}$ function calls to solve the shooter localization problem. The proposed CF method required an average of $5.9\times 10^{3}$ consensus function calls in the shooter test database, being the most efficient from among the consensus-based approaches. Note that the CF method guarantees that the global maximum is found, while the global convergence of other methods is not guaranteed. 

## 5. Conclusions

A fast and reliable calculation method has been proposed to find the maximum of the consensus function on a finite grid. The algorithm has been theoretically proven to find the global maximum of the consensus function on the grid. The proposed CF method is capable of solving the hyperbolic localization problem even in the presence of a large number of outlier measurements (e.g., from NLOS measurements). The method is extremely robust: it provides estimates with an error level close to the theoretical optimum (Cramer–Rao lower bound) when a large percentage (even as much as 70%) of the measurements are outliers. 

The application of the CF method is beneficial in situations where measurements include outliers (e.g., NLOS measurements or bad sensor detections), even a large number of them, and the necessary level of accuracy is still near the theoretical minimum. One disadvantage of the CF method is that its computational cost is higher than that of the simple LS method. Also, its speed depends on the actual error surface; thus, it cannot be predicted and guaranteed. Therefore, the application of the CF method is suggested in soft real-time systems. 

The consensus-based approach is not limited to TDOA measurements only. The derivation of hybrid consensus functions including TDOA and angle of arrival (AOA) or angle difference of arrival (ADOA) measurements requires further research. Another topic for future work is the more efficient implementation of the proposed method through the use of parallel implementation.

## Figures and Tables

**Table 1:** Target positions in the simulation experiment.

ID	*x* (m)	*y* (m)	*z_2D_* (m)	*z_3D_* (m)
1	20	50	0	1.5
2	30	30	0	3.1
3	40	10	0	0.5
4	30	60	0	0.2
5	40	40	0	1.1
6	50	20	0	0.1

**Table 2:** Results of the 2D simulation experiment with measurement noise of σ=0.1 m and no outlier measurements.

	N=15				N=25				N=35			
		RMSE (m)				RMSE (m)				RMSE (m)		
ID	$\sqrt{CRLB}$	LS	COMW	CF	$\sqrt{CRLB}$	LS	COMW	CF	$\sqrt{CRLB}$	LS	COMW	CF
1	0.071	0.103	0.086	0.077	0.061	0.090	0.076	0.068	0.048	0.084	0.063	0.060
2	0.068	0.092	0.091	0.077	0.051	0.073	0.057	0.057	0.044	0.079	0.052	0.059
3	0.082	0.092	0.099	0.090	0.067	0.076	0.089	0.072	0.054	0.078	0.067	0.063
4	0.065	0.114	0.098	0.067	0.053	0.083	0.077	0.060	0.047	0.082	0.064	0.059
5	0.064	0.083	0.080	0.065	0.048	0.086	0.061	0.064	0.042	0.078	0.054	0.062
6	0.068	0.072	0.071	0.073	0.057	0.071	0.088	0.064	0.046	0.074	0.057	0.059
mean	0.070	0.092	0.088	0.075	0.056	0.080	0.075	0.064	0.047	0.079	0.060	0.060

**Table 3:** Results of the 3D simulation experiment with measurement noise of σ=0.1 m and no outlier measurements.

	N=15				N=25				N=35			
		RMSE (m)				RMSE (m)				RMSE (m)		
ID	$\sqrt{CRLB}$	LS	COMW	CF	$\sqrt{CRLB}$	LS	COMW	CF	$\sqrt{CRLB}$	LS	COMW	CF
1	0.27	0.32	0.30	0.27	0.20	0.25	0.21	0.21	0.18	0.26	0.22	0.22
2	0.13	0.15	0.14	0.14	0.11	0.13	0.11	0.11	0.10	0.11	0.11	0.10
3	0.21	0.24	0.33	0.25	0.14	0.15	0.19	0.14	0.12	0.15	0.16	0.12
4	0.30	0.43	0.30	0.45	0.23	0.45	0.23	0.41	0.21	0.46	0.27	0.39
5	0.16	0.19	0.17	0.16	0.10	0.14	0.12	0.12	0.09	0.13	0.12	0.11
6	0.20	0.21	0.22	0.32	0.12	0.14	0.15	0.17	0.10	0.13	0.13	0.15
mean	0.21	0.26	0.24	0.27	0.15	0.21	0.17	0.19	0.13	0.21	0.17	0.18

**Table 4:** Results of the 2D simulation experiment with measurement noise of σ=0.1 m and $N_{out}=1\ldots 6$ outlier measurements.

	$N=15,\;1\leq N_{out}\leq 3$				$N=25,\;1\leq N_{out}\leq 4$				$N=35,\;1\leq N_{out}\leq 6$			
		RMSE (m)				RMSE (m)				RMSE (m)		
ID	$\sqrt{CRLB}$	LS	COMW	CF	$\sqrt{CRLB}$	LS	COMW	CF	$\sqrt{CRLB}$	LS	COMW	CF
1	0.071	3.5	3.5	0.13	0.061	2.2	3.6	0.20	0.048	1.7	3.4	0.20
2	0.068	3.4	1.9	0.14	0.051	1.9	1.2	0.12	0.044	3.2	1.1	0.13
3	0.082	3.5	3.8	0.16	0.067	2.8	4.8	0.22	0.054	4.4	3.1	0.15
4	0.065	2.2	3.8	0.22	0.053	1.6	4.0	0.19	0.047	1.9	4.8	0.37
5	0.064	4.4	1.5	0.13	0.048	2.3	0.8	0.12	0.042	2.3	0.7	0.14
6	0.068	2.7	2.9	0.38	0.057	2.9	2.1	0.47	0.046	1.9	1.5	0.15
mean	0.070	3.3	2.9	0.19	0.056	2.3	2.8	0.22	0.046	2.6	2.4	0.19

**Table 5:** Results of the 3D simulation experiment with measurement noise of σ=0.1 m and $N_{out}=1\ldots 6$ outlier measurements.

	$N=15,\;1\leq N_{out}\leq 3$				$N=25,\;1\leq N_{out}\leq 4$				$N=35,\;1\leq N_{out}\leq 6$			
		RMSE (m)				RMSE (m)				RMSE (m)		
ID	$\sqrt{CRLB}$	LS	COMW	CF	$\sqrt{CRLB}$	LS	COMW	CF	$\sqrt{CRLB}$	LS	COMW	CF
1	0.27	16	9.1	0.40	0.20	4.8	2.7	0.37	0.18	5.2	2.6	0.27
2	0.13	6.6	3.3	0.29	0.11	2.9	1.2	0.27	0.10	18.0	2.3	0.22
3	0.21	7.2	6.5	0.38	0.14	3.9	4.5	0.24	0.12	2.2	3.8	0.25
4	0.30	5.9	7.7	0.88	0.23	6.5	3.5	0.45	0.21	10.1	5.7	0.81
5	0.16	5.7	3.7	0.19	0.10	5.2	2.3	0.44	0.09	4.3	0.95	0.17
6	0.20	9.8	5.4	0.84	0.12	2.6	3.1	0.25	0.10	4.7	1.7	0.37
mean	0.21	8.5	6.0	0.50	0.15	4.3	2.9	0.34	0.13	7.4	2.8	0.35

**Table 6:** Execution times of the LS, COM-W, and CF methods in the simulation experiments.

N	Mean Execution Time, 2D (ms)			Mean Execution Time, 3D (ms)		
	LS	COM-W	CF	LS	COM-W	CF
15	3.9	34.8	18.8	5.0	95.6	89.0
25	3.7	164.8	19.8	4.7	814.7	85.8
35	3.6	439.5	20.8	4.7	3225.7	83.1
mean	3.7	213.1	19.8	4.8	1378.7	86.0

**Table 7:** Estimation errors and execution times of the LS, COM-W, and CF methods in the indoor localization experiment.

	RMSE-xy (m)	RMSE-xyz (m)	Execution Times (ms)
LS	0.24	0.62	4.2
COM-W	0.19	0.47	4.3
CF	0.15	0.41	11.1

**Table 8:** Source positions in the counter-sniper experiment.

ID	*x* (m)	*y* (m)	*z* (m)
1	36.34	67.55	3.55
2	30.30	66.42	−0.30
3,4	31.94	57.34	−0.30
5,6,7,8	28.93	45.45	7.30
9	25.85	40.44	−0.20
10	33.37	48.24	−0.25

**Table 9:** Shooter position estimation errors along with the number *N* of sensors providing measurements and the consensus function value *C_w_*.

ID	Position Estimation Error (m)			Run-Time (ms)			*N*	*C_w_*
	LS	COM-W	CF	LS	COM-W	CF		
1	14.22	10.04	0.80	105	1404	109	29	25
2	2.34	2.74	1.28	24	11	350	10	9
3	1.50	2.00	1.18	6	69	52	26	23
4	4.65	3.91	0.89	6	2261	92	34	25
5	12.41	8.79	0.34	8	2789	60	38	23
6	3.29	6.13	0.14	6	292	141	21	19
7	13.00	12.51	0.50	4	215	490	20	16
8	3.54	3.37	0.77	5	67	125	15	14
9	7.82	11.37	1.06	6	1159	34	30	21
10	4.82	5.32	1.94	6	1224	79	30	22
average	6.76	6.62	0.89	18	949	153		

## Data Availability

The UTIL Ultra-wideband Dataset used in this study is available at the following public domain database: https://utiasdsl.github.io/util-uwb-dataset/ (accessed on 7 February 2004).

## List of Symbols

List and definition of symbols.

**Notations**	**Definition**
c	propagation speed
$C_{w}$	consensus function with window w
${\bar{C}}_{w}$	$\mathrm{upper}\;\mathrm{bound}\;\mathrm{of}\;C_{w}$
D	dimension of the search space
$d_{i}\left(P\right)=d_{i}\left(x,y,z\right)$	distance between P$\;\mathrm{and}\;S_{i}$
$G_{c}$	coarse grid
$G_{f}$	fine grid
$P_{0}=\left(x_{0},\;y_{0},\;z_{0}\right)$	source position
$p_{i}=\left(x_{i},y_{i},z_{i}\right)$	position of sensor #i
$S_{i}$	sensor #i
$T_{0}$	emission time
$T_{i}\left(P\right)=T_{i}\left(x,y,z\right)$	$\mathrm{emission}\;\mathrm{time}\;\mathrm{estimated}\;\mathrm{from}\;t_{i}$, provided the source is at P
$t_{i}$	detection time at sensor #i
$d_{i}\left(P\right)=d_{i}\left(x,y,z\right)$	distance between P$\;\mathrm{and}\;S_{i}$
w	window length
$\left({\hat{x}}_{0},{\hat{y}}_{0},{\hat{z}}_{0}\right)\;$	location estimate
Δ	$\mathrm{grid}\;\mathrm{size}\;\mathrm{of}\;G_{f}$
Δs	maximum sensor location error
Δτ	maximum time measurement error
$\Lambda_{w}$	window function
$\Psi_{w}$	set of points where the consensus function takes its maximum
